# Diagnostic Accuracy of Strain Ultrasound Elastography in Thyroid Lesions Compared to Fine-Needle Aspiration Cytology

**DOI:** 10.7759/cureus.27185

**Published:** 2022-07-23

**Authors:** Aniqua Saleem, Umme Kalsoom, Sundas Yasin, Misbah Durrani, Saba Akram, Riffat Mushtaq

**Affiliations:** 1 Radiology Department, Holy Family Hospital, Rawalpindi Medical University, Rawalpindi, PAK; 2 Radiology Department, Benazir Bhutto Hospital, Rawalpindi Medical University, Rawalpindi, PAK; 3 Radiology Department, Rawalpindi Institute of Cardiology, Rawalpindi, PAK; 4 Radiology Department, Avicenna Medical College, Lahore, PAK; 5 Radiology Department, District Head Quarter Hospital, Mirpur Azad Kashmir, PAK

**Keywords:** negative predictive value, positive predictive value, fine needle aspiration, thyroid nodules, sensitivity, ultrasound elastography

## Abstract

Introduction

Strain ultrasound-guided elastography (USE) could be used to differentiate malignant from benign thyroid lesions if its sensitivity and specificity are significantly high. Data on whether to rely on USE in differentiating thyroid nodules are unavailable, and fine-needle aspiration cytology (FNAC) remains the gold standard. However, FNAC carries a significant financial burden on hospitals and psychological stress on patients. Therefore, we conducted this study to determine the diagnostic accuracy of strain USE in thyroid lesions.

Methodology

We conducted a descriptive cross-sectional study at the Radiology Department, Benazir Bhutto Hospital, Rawalpindi, from December 6, 2020, to June 5, 2021. The study included adult patients aged between 20 to 70 years who were referred with thyroid nodules or lesions found clinically or on routine neck ultrasound. The study excluded patients who had previous history of surgery or previously diagnosed with malignant thyroid lesions and recurrent thyroid nodules. Strain USE was performed on thyroid nodules, and the degree of strain was color-coded on a scale from red (soft, greatest elasticity) to green (intermediate, average strain) to blue (hard, no elasticity/strain). Lesions were given an elasticity score on a five-point scale. The lesion was given a score of one if the entire lesion was uniformly shaded in green. A lesion with mosaic pattern of green and blue was scored as two. A score of three denoted a lesion with green periphery and blue center on strain elastography. A score of four indicated uniform blue in the entire lesion, with green in the lesion's periphery. The highest score of five was given if the lesion and its surroundings demonstrated blue color. Ultrasound-guided FNAC of the thyroid nodules was performed following USE. Data was analyzed using IBM Corp. Released 2011. IBM SPSS Statistics for Windows, Version 20.0. Armonk, NY: IBM Corp. Mean ± standard deviation for calculating quantitative variables. Frequencies and percentages were calculated for qualitative variables. Sensitivity, specificity, positive predictive value (PPV), negative predictive value (NPV), and diagnostic accuracy of strain USE was calculated taking FNAC as gold standard. We also conducted a receiver operating characteristic curve analysis to quantify the diagnostic accuracy of strain USE in thyroid lesions.

Results

The study included 207 adult patients (117 women, 56.52%; 90 men, 43.48%). The study population's mean age was 50.0 ± 11.8 years (range, 20 to 70 years). Most patients (56.52%) were aged 46 to 70 years. FNAC confirmed malignant thyroid nodules in 100 cases (true positive), and nine cases (false positive) had no malignant lesions on FNAC. In USE-negative patients, 91 were true negative, while seven were false negative. Strain USE's overall sensitivity was 93.46%, specificity was 91.0%, PPV was 91.74%, NPV was 92.86%, and diagnostic accuracy was 92.27% compared to the gold standard FNAC.

Conclusions

Strain USE in thyroid lesions is a noninvasive modality of choice with high diagnostic accuracy and has dramatically improved our ability to diagnose malignant thyroid nodules preoperatively. Strain USE also helps the surgeons in proper decision-making. Strain USE should be used routinely in all patients with thyroid lesions to help diagnose malignant thyroid nodules preoperatively and inform proper surgical and treatment plans.

## Introduction

Thyroid nodules are very common, and their incidence is gradually increasing [1,2]. Most are benign; however, malignancy occurs in 5% to 15% of cases [1]. Thyroid nodules are usually discovered when patients present for physical examination after feeling an enlarged nodule in their neck or experiencing visible neck swelling. The clinician refers the patient for an ultrasound to look for thyroid lesions and characterize them according to the sonological criteria known as Thyroid Imaging Reporting and Data System (TIRADS). A benign nodular goiter is the most common pathology of the thyroid gland, and assessing and diagnosing malignancy in these goiters remains a challenge for radiologists. Elastography and two-dimensional sonography may help categorize individual thyroid nodules [3]. A thyroid hormonal profile, including thyroid-stimulating hormone, T3, and T4 levels, is also performed to categorize the nodules as functional or nonfunctional, which helps characterize nodules as benign or malignant. Similarly, serum calcitonin levels should also be measured to exclude medullary thyroid cancer [4]. The 2015 American Thyroid Association (ATA) guidelines state that the malignant risk of high-suspicion thyroid nodules is > 70% to 90% [5]. However, the ATA characteristics of high-suspicion thyroid nodules overlap with degrees 4a to 5 of Kwak's TIRADS, and the frequency of malignancy ranges from 3.3% to 87.5%. Therefore, using strain elastography to differentiate malignant from benign thyroid nodules of high suspicion could be fruitful [6]. A recent study by Okasha et al. recommended including strain elastography in TIRADS classification for discriminating benign from malignant nodules [7]. Furthermore, according to the sonographic picture, thyroid nodules that show low, intermediate, or high suspicion of malignancy and all nodules with suspicious clinical findings should undergo fine-needle aspiration cytology (FNAC) [4].

FNAC is considered the most valuable preoperative method for differentiating malignant from benign thyroid lesions. Previous studies also suggest that ultrasound is a practical, cheap, and widely used technique in differentiating benign from malignant, but ultrasound does not have high sensitivity, specificity, and accuracy in diagnosing malignant nodules. Also, FNAC could be nondiagnostic, requiring additional sessions [2,8].

Ultrasound-guided elastography (USE) is a noninvasive test used to assess a tissue's biomedical properties (e.g., elasticity) [1,8]. It is helpful in various diagnostic applications, including thyroid nodules. Different types of USE are available. Strain USE, also known as real-time elastography, is the most widely available type [1]. Its basic principle is that, upon compression, softer parts of tissue deform easier than harder ones. This degree of tissue distortion under external force can be recorded, and thus tissue stiffness/hardness can be measured [1,2]. Strain USE is a qualitative means of measuring tissue stiffness; however, semiquantitative analysis of tissue stiffness can be measured using strain ratio, where stiffness/strain of a target lesion is compared with a reference normal thyroid tissue using real-time elastography [1,9]. Benign tissue is softer, elastic, and more easily deformable than malignant tissue. USE helps in measuring tissue elasticity and therefore helps differentiate benign (soft) from malignant nodules (hard) [10]. FNAC is an excellent method for differentiating benign from malignant nodules, but it is minimally invasive, time-consuming, and expensive, with a risk of serious complications [2,10].

In contrast, USE is noninvasive, cheap, easy to use, less time-consuming than FNAC, and has no adverse effects [8]. We conducted this study to use strain USE as a diagnostic test in differentiating malignant from benign thyroid lesions, provided its sensitivity and specificity are significantly high. The diagnostic accuracy of USE for detecting thyroid lesions is controversial, and no recent study in our region has assessed the use of USE in differentiating thyroid nodules. This study assesses the diagnostic accuracy of USE in local settings, which could limit the use of FNAC, exerting a financial burden on hospitals and psychological stress on patients.

## Materials and methods

Department of Diagnostic Radiology, Benazir Bhutto Hospital, Rawalpindi, was utilized to conduct this descriptive and cross-sectional research from December 6^th^, 2020, to June 5^th^, 2021. The sample size of 207 cases has been calculated with a 95% confidence level, 10% desired precision, and taking the expected prevalence of malignant thyroid nodules, i.e., 15% with sensitivity of 92% and specificity of 15% of strain ultrasound elastography in diagnosing malignant thyroid nodules [1].

Non-probability as well as consecutive sampling was done for patients (20-70 years age) referred with thyroid nodule/lesion, found clinically or on routine ultrasound neck examination. The exclusion criteria for this research included those patients who had a previous history of surgery or were previously diagnosed with malignant thyroid lesions, patients with recurrent thyroid nodules, and those patients who were not available for follow-up.

Hospital ethical committee approval, as well as informed consent from the patient, was obtained. Strain ultrasound elastography was performed by a postgraduate resident under the supervision of a consultant radiologist. ESAOTE (Genoa, Italy) ultrasound machine, with elastography procedure capability, attached to a 5-13 MHz high-frequency linear transducer was used. Elastography was performed by at least two radiologists with little or no interobserver difference. USE was performed by gentle pressing up and down motion of the probe on the patient`s neck till optimal pressure is achieved, as shown by the pressure bar lateral to the elastogram. The degree of strain within the region of interest was given colors by using the scale from red (soft, greatest elasticity) to green (intermediate, average strain) to blue (hard, no elasticity/strain) [8]. The lesion was scored by Ueno and Itoh`s study [11,12]. Ultrasound-guided FNAC of thyroid nodule was performed on the same day. A consultant pathologist, who was kept blind to elastography results, evaluated cytopathology results.

Data were analyzed in IBM Corp. Released 2011. IBM SPSS Statistics for Windows, Version 20.0. Armonk, NY: IBM Corp. The age of patients was taken as a quantitative variable and was measured as mean ± SD. Gender of the patient, duration of symptoms, family history, radiation exposure history, occupational history, elastography report, and FNAC reports were taken as qualitative variables and was measured as frequencies and percentages. 2 x 2 table was used to present data for calculation of sensitivity, specificity, PPV, NPV, and accuracy. Data was stratified for age, gender, duration of symptoms, family history, and radiation exposure history. Post-stratification 2 x 2 table was generated to calculate sensitivity, specificity, PPV, NPV, and accuracy. Receiver operating characteristic (ROC) curve analysis was used to quantify the diagnostic accuracy of strain ultrasound elastography in thyroid lesions.

## Results

The study included 207 adult patients aged 20 to 70 (mean age, 50.0 ± 11.8 years). Most patients were aged 46 to 70 (n=117; 56.52%). One hundred seventeen participants were women (56.52%), and 90 were men (43.48%; female-to-male ratio: 1.3:1). The mean duration of the disease was 9.16 ± 3.87 months. Less than one-third (30.92%) of patients had a positive family history, while 20.29% had X-ray radiation exposure.

All the patients were subjected to strain USE. USE supported the diagnosis of malignant thyroid nodules in 109 patients. FNAC confirmed malignant thyroid nodules in 100 cases (i.e., true positive), whereas nine had no malignant lesion on FNAC (i.e., false positive). In USE-negative patients, 91 were true negative, while seven were false negative (p<.0001; Table 1).

**Table 1:** Diagnostic accuracy of strain USE in thyroid lesions keeping FNAC as the gold standard USE: Ultrasound elastography; FNAC: Fine-needle aspiration cytology; PPV: Positive predictive value; NPV: Negative predictive value.

Parameter	Percent
Sensitivity	93.46%
Specificity	91.0%
PPV	91.74%
NPV	92.86%
Diagnostic Accuracy	92.27%

**Figure 1:**
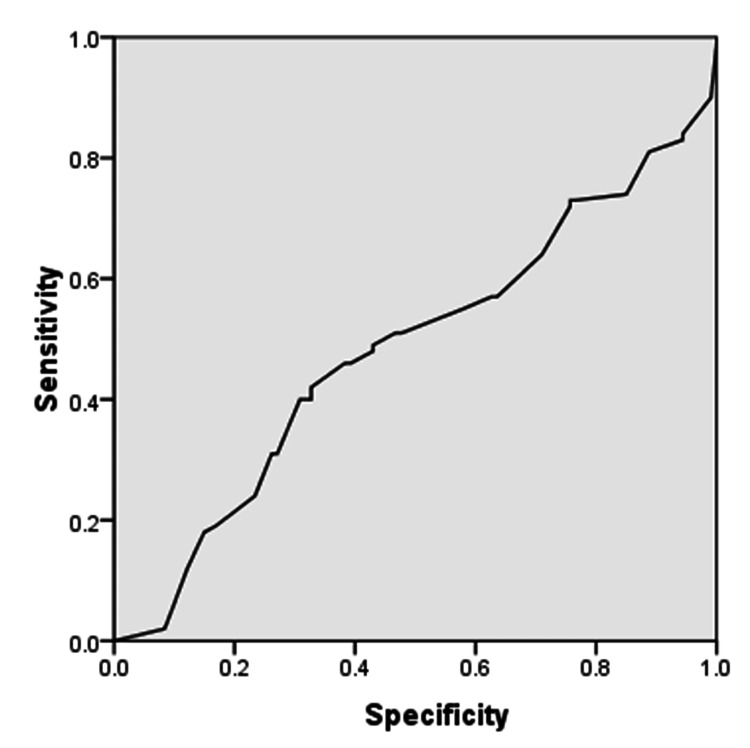
ROC curve

For strain USE in thyroid lesions, the overall sensitivity was 93.46%, specificity was 91.0%, PPV was 91.74%, NPV was 92.86%, and diagnostic accuracy was 92.27% against FNAC as the gold standard. The ROC curve is shown in Figure 1.

Table 2 and Table 3 present the stratification of diagnostic accuracy concerning age groups, and Table 4 and Table 5 present the stratification according to sex. Similarly, the stratification of diagnostic accuracy concerning disease duration is presented in Table 6 and Table 7, and positive family history is presented in Table 8 and Table 9. Finally, positive radiation exposure is given in Table 10 and Table 11.

**Table 2:** Stratification of USE diagnostic accuracy according to age USE: Ultrasound elastography; PPV: Positive predictive value; NPV: Negative predictive value.

Age Group (Years)	Parameter	Percent	P-Value
20 to 45 (n=90)	Sensitivity	95.74%	0.001
	Specificity	93.02%	
	PPV	93.75%	
	NPV	95.24%	
	Diagnostic Accuracy	94.44%	
46 to 70 (n=117)	Sensitivity	91.67%	0.001
	Specificity	89.47%	
	PPV	90.16%	
	NPV	91.07%	
	Diagnostic Accuracy	91.60%	

**Table 3:** Stratification of USE diagnostic accuracy by age compared to FNAC results USE: Ultrasound elastography; FNAC: Fine-needle aspiration cytology; TP: True positive; FP: False positive; TN: True negative; FN: False negative.

Age Group (years)	USE	Positive on FNAC	Negative on FNAC	P-Value
20 to 45 (n=90)	USE Positive Result	45 (TP)	03 (FP)	0.001
	USE Negative result	02 (FN)	40 (TN)	
46 to 70 (n=117)	USE Positive Result	55 (TP)	06 (FP)	0.001
	USE Negative result	05 (FN)	51 (TN)	

**Table 4:** Stratification of USE diagnostic accuracy according to sex USE: Ultrasound elastography; PPV: Positive predictive value; NPV: Negative predictive value.

Sex	Parameter	Percent	P-value
Males (n=90)	Sensitivity	89.80%	0.001
	Specificity	92.68%	
	PPV	93.62%	
	NPV	88.37%	
	Diagnostic Accuracy	91.11%	
Females (n=117)	Sensitivity	96.55%	0.001
	Specificity	89.83%	
	PPV	90.32%	
	NPV	96.36%	
	Diagnostic Accuracy	93.16%	

**Table 5:** Stratification of USE diagnostic accuracy by sex compared to FNAC results USE: Ultrasound elastography; FNAC: Fine-needle aspiration cytology; TP: True positive; FP: False positive; TN: True negative; FN: False negative.

Sex	USE	Positive on FNAC	Negative on FNAC	P-Value
Males (n=90)	USE Positive Result	44 (TP)	03 (FP)	0.001
	USE Negative result	05 (FN)	38 (TN)	
Females (n=117)	USE Positive Result	56 (TP)	06 (FP)	0.001
	USE Negative result	02 (FN)	53 (TN)	

**Table 6:** Stratification of USE diagnostic accuracy according to disease duration USE: Ultrasound elastography; PPV: Positive predictive value; NPV: Negative predictive value.

Disease Duration	Parameter	Percent	P-value
≤ 9 months (n=138)	Sensitivity	91.67%	0.001
	Specificity	90.91%	
	PPV	91.67%	
	NPV	90.91%	
	Diagnostic Accuracy	91.30%	
> 9 months (n=117)	Sensitivity	91.14%	0.001
	Specificity	91.18%	
	PPV	91.89%	
	NPV	96.88%	
	Diagnostic Accuracy	94.20%	

**Table 7:** Stratification of USE diagnostic accuracy by disease duration compared to FNAC results USE: Ultrasound elastography; FNAC: Fine-needle aspiration cytology; TP: True positive; FP: False positive; TN: True negative; FN: False negative.

Disease Duration	USE	Positive on FNAC	Negative on FNAC	P-Value
≤ 9 months (n=138)	USE Positive Result	66 (TP)	06 (FP)	0.001
	USE Negative result	06 (FN)	60 (TN)	
Females (n=117)	USE Positive Result	34 (TP)	03 (FP)	0.001
	USE Negative result	01 (FN)	31 (TN)	

**Table 8:** Stratification of USE diagnostic accuracy according to family history USE: Ultrasound elastography; PPV: Positive predictive value; NPV: Negative predictive value.

Family History	Parameter	Percent	P-value
Positive (n=64)	Sensitivity	97.22%	0.001
	Specificity	89.29%	
	PPV	92.11%	
	NPV	96.15%	
	Diagnostic Accuracy	93.75%	

**Table 9:** Stratification of USE diagnostic accuracy by family history compared to FNAC results USE: Ultrasound elastography; FNAC: Fine-needle aspiration cytology; TP: True positive; FP: False positive; TN: True negative; FN: False negative.

Family History	USE	Positive on FNAC	Negative on FNAC	P-Value
Positive (n=64)	USE Positive Result	35 (TP)	03 (FP)	0.001
	USE Negative result	01 (FN)	25 (TN)	

**Table 10:** Stratification of USE diagnostic accuracy according to radiation exposure USE: Ultrasound elastography; PPV: Positive predictive value; NPV: Negative predictive value.

Radiation Exposure	Parameter	Percent	P-value
Positive (n=42)	Sensitivity	95.83%	0.001
	Specificity	88.89%	
	PPV	92.00%	
	NPV	94.12%	
	Diagnostic Accuracy	92.86%	

**Table 11:** Stratification of USE diagnostic accuracy by radiation exposure compared to FNAC results USE: Ultrasound elastography; FNAC: Fine-needle aspiration cytology; TP: True positive; FP: False positive; TN: True negative; FN: False negative.

Radiation Exposure	USE	Positive on FNAC	Negative on FNAC	P-Value
Positive (n=42)	USE Positive Result	23 (TP)	02 (FP)	0.001
	USE Negative result	01 (FN)	16 (TN)	

## Discussion

Thyroid nodules are frequent, and sonography is an economical, widely available modality free from potential radiation hazards; sonography could be used for diagnosing thyroid nodules. Various sonographic features like shape (e.g., elliptical, taller-than-wide, irregular), margins (e.g., lobulated, spiculated, poorly marginated), and presence of calcification (e.g., noncalcified, microcalcification, macrocalcification, crystals) aid in categorizing the thyroid nodule as benign or malignant. Furthermore, a Doppler assessment contributes by indicating the presence of color flow within or at the lesion's periphery. The elastography component of ultrasound provides information on the stiffness of the lesion; the stiffer the tissue, the more likely it is malignant [12]. We conducted this study to determine the diagnostic accuracy of strain USE in thyroid lesions against the gold standard of FNAC.

In Pakistan, the accuracy of FNAC approaches 95% in the best centers, with sensitivity ranging from 65% to 98% and specificity from 72% to 100% [13]. The overall sensitivity and specificity of strain USE in thyroid lesions against FNAC as the gold standard was 93.46% and 91.0%, respectively. This was higher than the results from Cabanillas et al., who reported strain USE sensitivity and specificity of 77% and 85%, respectively [4]. 

In 2015, Cantisani et al. conducted a meta-analysis of 10 studies which showed that the mean sensitivity of elastography was 92%, and the mean specificity was 90% in detecting malignancy within a thyroid nodule [1]. They reported that elastography in conjunction with ultrasound enhances accuracy for detecting thyroid malignancy, thus reducing the risk of diagnosing a large number of nodules via invasive FNAC. While FNAC is the gold standard, USE can help identify nodules that need FNAC, and it can be a follow-up procedure to confirm benign lesions on FNAC [1]. There are six Bethesda categories for thyroid fine needle aspirates; the first three of them, Bethesda I nondiagnostic, Bethesda II benign, and Bethesda III atypia of undetermined significance are somewhat non-conclusive. Here comes the role of USE which may help in reducing the number of unnecessary FNACs. A previous meta-analysis by Hairu et al. showed that the use of USE reduces the frequency of FNAC by 33%-77% [5].

Another meta-analysis conducted in 2013 by Razavi et al. compared 24 studies and concluded that the diagnostic performance of elastography was better than brightness-mode ultrasound [14]. All studies in the meta-analyses by Cantisani et al. and Razavi et al. showed similar results except for one study by Moon et al. They studied 703 nodules and calculated sensitivity to be 65% and specificity as 58% in detecting thyroid nodule malignancy by elastography [15]. This discrepancy in Moon et al.'s results was attributed to the difference in thyroid malignancy prevalence in the study population and because Moon et al. excluded complex lesions and lesions with macrocalcifications, altering the results [1,14,15]. Cantisani et al. also studied different scoring and color-coding systems, concluding that the elastography color scoring used by Itoh et al. for breast elastography was also feasible for grading thyroid lesions [1,11]. In 2014, Ghajarzadeh et al. conducted a meta-analysis of 12 studies exploring the threshold for elasticity scores. They concluded that the score threshold for characterizing a nodule as malignant is two to three, with a combined sensitivity of 92% and a specificity of 90% [16].

Abdelrahman et al. studied 73 indeterminate thyroid nodules and found that 16 nodules were malignant and 57 were benign. On USE, all 57 nodules diagnosed as benign had a score of one to three, while 15 of 16 (93.75%) diagnosed as carcinoma had scores of four to five, with 93.3% sensitivity, 100% specificity, and 97.8% accuracy [17]. USE revealed that hypoechogenicity scores of four to five were most predictive of malignancy with a sensitivity of 80%, specificity of 100%, and accuracy of 93.4%. The strain ratio cutoff value for malignant nodules was determined as 2.3 [17]. Five nodules of 16 had SR between 2.31 and four (sensitivity was 96% and specificity was 83%) [17].

Asteria et al. reported that elasticity scores of four to five were highly predictive of malignancy, with a sensitivity of 90.63%, a specificity of 89.47%, and an accuracy of 90.20%. They reported that the sensitivity, specificity, PPV, and NPV of USE for thyroid cancer diagnoses were 94.1%, 81%, 55.2%, and 98.2%, respectively, while the accuracy was 83.7% [18].

Elawa et al. demonstrated that malignant nodules had a statistically significantly higher degree of color and strain ratio than benign nodules (p<0.05). Nodules with an elastography score of two were benign, while those with elastography scores of four and five were mostly malignant. The best strain ratio cutoff value to differentiate benign from malignant nodules was 2.90, with 86.4% sensitivity, 90.3% specificity, PPV of approximately 61.3%, NPV of approximately 97.4%, and accuracy of approximately 89.7% [19].

Kagoya et al. concluded that a strain ratio or strain index value greater than 1.5 is a predictor of thyroid malignancy and exhibits 90% sensitivity and 50% specificity, which is in concordance with our results as we found a strain index higher than 1.6 was an independent predictor of thyroid malignancy, with sensitivity and specificity of 89% and 70%, respectively [20]. Sun et al. studied 5481 nodules in 4468 patients for elasticity scores and 1063 nodules in 983 patients for strain ratios. The overall mean sensitivity and specificity of USE for differentiating thyroid nodules were 0.79 (95% confidence interval [CI], 0.77-0.81) and 0.77 (95% CI, 0.76-0.79) for the elasticity score assessment and 0.85 (95% CI, 0.81-0.89) and 0.80 (95% CI, 0.77-0.83) for the strain ratio assessment. The areas under the curve for the elasticity score and strain ratio were 0.8941 and 0.9285, respectively [21].

Overall, our results are supported by previous studies in the literature. Strain USE is the noninvasive modality of choice with high diagnostic accuracy in diagnosing malignant thyroid nodules and has dramatically improved our ability to diagnose malignant thyroid nodules preoperatively and helps surgeons make proper decisions.

Our study had several limitations. We did not combine and correlate our study with gray scale ultrasound features and with TIRADS classification. A recent study suggests that combining TIRADS, strain elastography results, and apparent diffusion coefficient (ADC) values of thyroid nodules may produce promising noninvasive results [22]. The combination of American College of Radiology-TIRADS, USE, and ADC value from magnetic resonance imaging may add valuable data and increase the sensitivity [22]. Secondly, lesion depth and thyroid parenchymal density could affect lesion elasticity, and these parameters should have been studied concurrently to avoid bias. Furthermore, carotid artery pulsations could create variable tissue deformations, and we should have studied the effect of nodule distance from the carotid artery. Finally, the histological features of fibrosis in thyroiditis or within calcified cysts and nodules within a multinodular goiter could alter the elastography results; this relationship should be further explored.

## Conclusions

Strain USE in thyroid lesions is the noninvasive modality of choice with high diagnostic accuracy. We suggest that thyroid lesions should be routinely assessed in a similar way to triple assessment in breast lesions, that is clinical assessment, radiological imaging, and pathological assessment. In thyroid imaging, USE should be used in conjunction with gray scale USG. We suggest that nodules with TIRADS score up to 2 and USE score of 1 and 2 should only be followed up clinically and by gray scale USG in combination with USE; however, a thyroid nodule with USE score of 3 and above should proceed for pathological examination. However, we do not recommend following up on a thyroid nodule with USE if there is clinical suspicion of thyroiditis or patients who had a previous history of FNAC or thyroid surgery. Strain USE has dramatically improved our ability to diagnose malignant thyroid nodules preoperatively and helps surgeons make proper decisions. Therefore, strain USE should be conducted routinely in all thyroid lesion patients for accurate diagnosis of malignant thyroid nodules preoperatively and to inform proper surgical and treatment planning.
